# Diagnosis of Encephalopathy Based on Energies of EEG Subbands Using Discrete Wavelet Transform and Support Vector Machine

**DOI:** 10.1155/2018/1613456

**Published:** 2018-07-02

**Authors:** Jisu Elsa Jacob, Gopakumar Kuttappan Nair, Thomas Iype, Ajith Cherian

**Affiliations:** ^1^Department of Electronics and Communication Engineering, SCT College of Engineering, Thiruvananthapuram, Kerala, India; ^2^Department of Electronics and Communication Engineering, TKM College of Engineering, Kollam, Kerala, India; ^3^Department of Neurology, Government Medical College, Thiruvananthapuram, Kerala, India; ^4^Department of Neurology, SCTIMST, Thiruvananthapuram, Kerala, India

## Abstract

EEG analysis in the field of neurology is customarily done using frequency domain methods like fast Fourier transform. A complex biomedical signal such as EEG is best analysed using a time-frequency algorithm. Wavelet decomposition based analysis is a relatively novel area in EEG analysis and for extracting its subbands. This work aims at exploring the use of discrete wavelet transform for extracting EEG subbands in encephalopathy. The subband energies were then calculated and given as feature sets to SVM classifier for identifying cases of encephalopathy from normal healthy subjects. Out of various combinations of subband energies, energy of delta subband yielded highest performance parameters for SVM classifier with an accuracy of 90.4% in identifying encephalopathy cases.

## 1. Introduction

Electroencephalogram (EEG) is a signal which represents the electrical activity of millions of neurons in the brain. The signal is acquired from the surface of the scalp. Since it reflects the neuronal activity of the cerebral cortex, it is used in the diagnosis of diseases which involves the function of the cortical neurons. The “EEG picture” of a disease is often a visual waveform or an abnormal frequency or a hypersynchrony or abnormalities in waveform amplitude.

EEG signals are nonstationary; i.e., the frequency components present in the signal vary with time [1]. Therefore, time domain and frequency domain analysis are not sufficient to give information of such signals. The time domain features included the signal statistics like power of the signal, mean, standard deviation, etc. First difference and second difference are also computed in time series analysis to get the signal variation over time [2]. Another time domain feature, namely, Normalized Length Density, was proposed by Jenke et al., which quantifies self-similarities within the EEG signal [3]. Another time domain feature for EEG analysis was proposed by Hausdorff et al. is the Nonstationary Index (NSI) [4]. The NSI gives a measure of the stationarity of the signal and measures the variation of segments average over time [2]. Frequency domain analysis includes employing fast Fourier transform (FFT) to calculate power spectrum of signal, relative power of EEG subbands, etc. [5, 6]. But none of these methods can completely visualise the nonstationarity behaviour of the signal.

Biomedical signals can be analysed in a better manner using time-frequency analysis [7–9]. Wavelet transforms give the information of various frequency components present in the signal at various instants. Thus, more information about EEG subbands can be extracted by employing wavelet transform instead of Fourier transform which is a frequency domain approach. Many other studies have reported the use of discrete wavelet transform (DWT) in the analysis of EEG, most popularly in epilepsy [10–14] and Alzheimer's disease [15, 16]. Discrete wavelet transform (DWT) has been widely used in the processing and analysis of biomedical signals as they are nonstationary. DWT has a major advantage over Fourier transform as it can obtain both time and frequency information from the signal. Wavelet decomposition of a time series signal is obtained by passing it through a series of high pass and low pass filters. The advantage for DWT is the low computation time and ease of implementation.

In our study, we have explored the application of discrete wavelet transform in EEG analysis in cases of encephalopathies. Encephalopathy is a disease of the brain due to malfunction or structural changes resulting from metabolic disorders due to organ dysfunction, chemicals, medications, or injuries [17]. The common causes are hyponatremia, hepatic failure, renal failure, carbon dioxide narcosis, and sepsis. Normal function of the brain depends on the normal neuronal metabolism which is related to the systemic homeostasis of the metabolites like glucose, electrolytes, amino acids, etc. The failure of major organs like kidneys, liver, lungs, and the respiratory system may result in a general deterioration of the brain function. This type of secondary impairment of the brain due to the malfunctioning of other organs is called “metabolic encephalopathy” [18]. We have compared the energies of the subbands, which were obtained using DWT, with that of normal and classified using support vector machine (SVM).

Demir et al. observed reduction in the alpha, asynchronous slow waves, focal slow activities, triphasic waves, burst-suppression pattern, and generalized or focal spike-sharp activities in encephalopathic EEG [18]. Spectral analysis of EEG of patients with hepatic encephalopathy was done by Amodio et al. by calculating mean dominant frequency (MDF) and relative powers of subbands using fast Fourier transform and autoregressive modelling [5]. Sagales et al. reported combination of a decreased alpha power spectral density, increased delta power spectral density, and decreased mean dominant frequency as a good feature set for discriminating hepatic encephalopathy cases from normal group using frequency domain analysis [19]. Amodio et al. applied an artificial neural network-expert system procedure (ANNES) with visual and spectral analysis of EEG to identify cases of hepatic encephalopathy [20]. Demir et al. concluded that there is no significant correlation between EEG findings and 2 groups of encephalopathy and normal healthy control [18]. However, to the best of our knowledge, the results of applying wavelet transform for analysing EEG in patients of encephalopathy are not reported till date.

## 2. Materials and Methods

### 2.1. Data Collection

The EEG data needed for this analysis was collected from patients of encephalopathy and healthy individuals from EEG lab of Neurology Department, Government Medical College, Thiruvananthapuram, Kerala. We studied a sample consisting of 232 EEG epochs of 15 encephalopathic patients and 218 EEG epochs of 12 normal healthy subjects. Encephalopathic cases included hepatic and uremic encephalopathy.

Patients with structural pathology, infections of the CNS, and cerebral vascular insult (confirmed by neuroimaging or other investigations) and patients with clinical picture suggestive of metabolic encephalopathy but without obvious metabolic disturbances detected in the necessary biochemical investigations and metabolic encephalopathy occurring in the background of another neurological illness causing cognitive dysfunction or a degenerative condition were excluded from our study. Normal healthy controls of the study include patients with single episode of syncope, who are clinically found to be normal and whose seizures and structural lesions were ruled out.

EEG epochs of 12-second duration were saved, from the artefact-free region of the recording under the supervision of two neurologists. EEG signals were recorded in EEG machine using NicVue software, in international 10-20 electrode system with 21-channel recording with average reference montage setting.

### 2.2. Mathematical Concepts of the Methodology

#### 2.2.1. LPF-TVD Denoising

**Figure 1:**
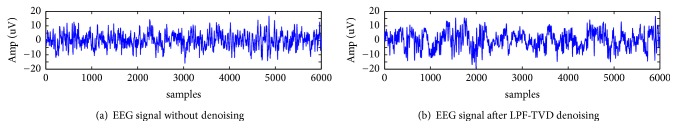
(a) Raw EEG signal without denoising; (b) EEG signal after LPF-TVD denoising.

This is a novel approach proposed by Selesnick et al. combining low pass filtering and sparse filtering [21]. Linear time invariant filtering, specifically low pass filtering (LPF), is applied for signals limited to a particular frequency band. Total variation denoising (TVD) is the method of estimating denoised signal from a noisy signal having a sparse derivative. As EEG signal consists of both low frequency components and sparse-derivative components, LPF-TVD approach is effective in preprocessing.  Figure 1 shows a raw EEG signal and the same signal after LPF-TVD filtering.

It is done by formulating the l1 norm of derivative of x which represents the signal having a sparse derivative. Noisy signal is represented by y=x+w. The optimization problem can be written as

(1) $$\begin{array}{c} \arg\underset{x}{\min}\left\{\;\frac{1}{2}{\left\|\;y-x\;\right\|}_{2}^{2}+\lambda {\left\|\;Dx\;\right\|}_{1}\;\right\} \end{array}$$

As approximation of derivative is the first-order difference; thus minimization of ‖Dx‖_1_ is done in (1). Its solution can be written as

(2) $$\begin{array}{c} tvd\left(\;y,\lambda \;\right)=\arg\underset{x}{\min}\left\{\;\frac{1}{2}{\left\|\;y-x\;\right\|}_{2}^{2}+\lambda {\left\|\;Dx\;\right\|}_{1}\;\right\} \end{array}$$

Here, majorization-minimization algorithm (MM Algorithm) proposed by Figueiredo et al. [22] is used to solve this problem for TVD denoising. Regularization parameter *λ* is taken as 0.8 for LPF-TVD filtering. This technique was found to be effective in denoising biomedical signals as evidenced in ECG preprocessing by Ray et al. [23] and in EEG processing [24].

#### 2.2.2. Discrete Wavelet Transform (DWT)

The technique of time-frequency analysis has been utilised in EEG analysis in many studies. EEG of epileptic patients was analysed using DWT and transient features like epileptic spikes were identified in time-frequency domain [11]. Osak et al. decomposed EEG into its subbands using DWT and reported that entropy of subbands yields better performance for classifying between seizures and normal group [10]. Three classes normal, schizophrenia, and obsessive compulsive disorder were classified based on wavelet decomposition parameters of EEG signal by Hazarika et al. [25]. These studies report good results for DWT in the field of EEG processing.

**Figure 2:**
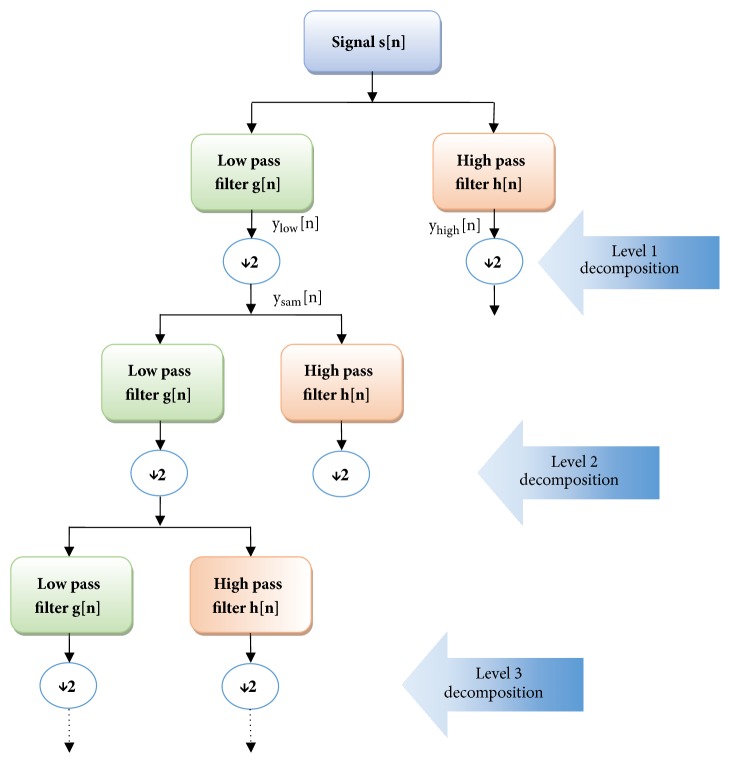
General structure of discrete wavelet transform.

DWT makes computation easier and faster by avoiding the redundant data which was processed in continuous wavelet transform. DWT of a signal s[n] was taken by passing it through a series of low pass and high pass filters to analyse low frequency and high frequency components, respectively (see Figure 2) [26, 27].

(3) ylown=sn∗gn=∑k=−∞∞sk.gn−k

(4) yhighn=sn∗hn=∑k=−∞∞sk.hn−k

Here, g[n] and h[n] represent the impulse response of low pass and high pass filters, respectively. The outputs of filters are given in (1) and (2). The low pass filter output samples are then downsampled by 2, thereby reducing the data redundancy (see (3)). As filter decreases the maximum frequency of signal from fm to fm/2, sampling frequency required is also decreased to half (as fs ≥ 2fm according to sampling theorem)[28]. Therefore, downsampling of the filter output will not result in any loss of information.

(5) $$\begin{array}{c} y_{sam}\left[\;n\;\right]=\overset{\infty }{\underset{k=-\infty }{\sum }}s\left[\;k\;\right].g\left[\;2n-k\;\right] \end{array}$$

After downsampling, sample number decreases to half and scale is doubled [29]. Thus, time-frequency information of EEG signal is obtained using DWT technique. All the analysis related to this was done using MATLAB R2014a software.

### 2.3. Processing of Data

The EEG epochs were subjected to a filtering process using a combined technique of low pass filtering and total variation denoising proposed by Ivan W Selesnick [21]. After denoising, wavelet decomposition was done to extract the subbands of EEG using DWT. The mother wavelet chosen for this work was Daubechies wavelet of order 4 (db4) because previous related works have pointed out the fact that its smoothing feature is appropriate for analysing EEG signals [30–34].

**Figure 3:**
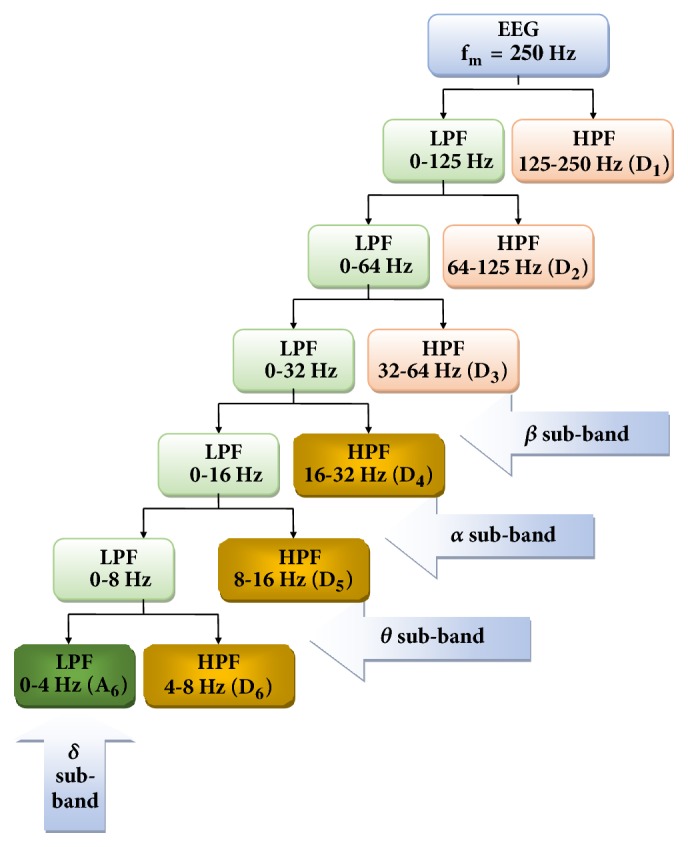
Application of DWT to generate subbands of EEG. Different levels of decomposition of DWT are shown. A6, D6, D5, and D4 yield delta, theta, alpha, and beta waves, respectively. fm: maximum frequency content of the EEG signal.

The EEG waves are conventionally classified into delta (less than 4 Hz), theta (4 to 7 Hz), alpha (8 to 13 Hz ), and beta waves (13 to 30 Hz), based on their frequency [35–37]. As the EEG epochs used for the study were recorded at a sampling frequency of 500Hz, maximum frequency content (fm) was taken to be 250Hz according to Nyquist sampling theorem. Based on this assumption, 6 levels of wavelet decomposition have been performed (refer to Figure 3). The approximation coefficients at 6th level (A6) and detailed coefficients at 6th level (D6), 5th level (D5), and 4th level (D4) yield delta, theta, alpha, and beta subbands of EEG, respectively.

Thus, the EEG was decomposed into its subbands and their energies were calculated from the wavelet coefficients [34, 38]. The relative energies of various subbands were calculated by dividing them with total energy of the signal (see (4) to (8)). Relative energies of the four subbands were utilised for classification.

Energy of delta subband:

(6) $$\begin{array}{c} E\left[\;A_{6}\;\right]=\overset{N}{\underset{j=1}{\sum }}{\left|\;A_{6}\;\right|}^{2} \end{array}$$

Energy of theta, alpha, and beta subbands:

(7) $$\begin{array}{c} E\left[\;D_{l}\;\right]=\overset{N}{\underset{j=1}{\sum }}{\left|\;D_{l}\;\right|}^{2}\;l=6,5,4 \end{array}$$

As *l*=6, total energy of EEG epoch is as follows:

(8) Etotal=EA6+∑j=16EDj

(9) Edelta=EA6Etotal

(10) Etheta/alpha/beta=EDiEtotali=6,5,4  for  theta, alpha, beta  respectively

**Figure 4:**
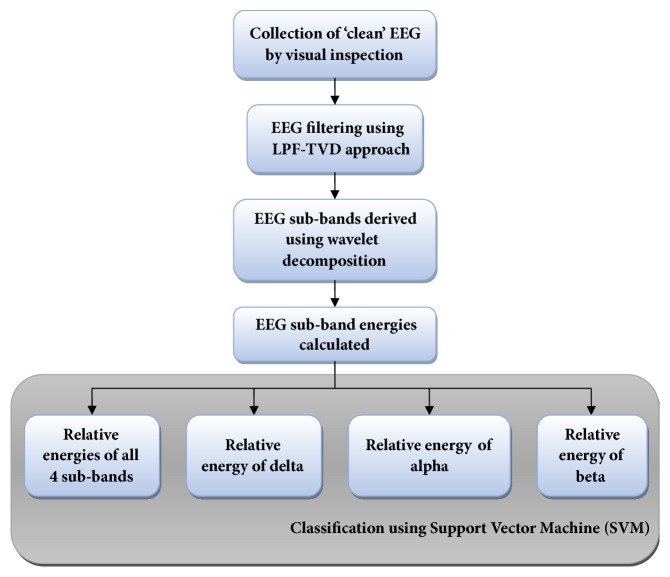
Flow diagram of the study.

Various subband energies (expressed as percentage of total energy called relative energy) were given as features to SVM for classifying EEGs of encephalopathic patients from that of normal healthy subjects. SVM is employed in our study as many studies reported good results for support vector machine (SVM) classification and even higher classification accuracy than neural networks in various neurological disorders [8, 39–41]. SVM is one of the commonly used methods for binary classification following supervised machine learning algorithm. It maps the data points to a higher dimensional space and tries to define a hyperplane separating the two classes with maximal margin [42, 43]. The kernel function that may be a linear, radial basis function (RBF), polynomial, or sigmoid kernel is responsible for the transformation to the higher dimensional space [43]. In this study, linear kernel function was used. Out of various inputs given to the classifier, the subband energy yielding higher performance parameters for identifying encephalopathy cases was found out. The flow diagram of this study is given in Figure 4.

## 3. Results and Discussion

### 3.1. Wavelet Analysis

Discrete wavelet transform (DWT) was performed on the data. EEG epochs of both normal and encephalopathic patients were decomposed into subbands, namely, delta, theta, alpha, and beta using DWT. Here, as the sampling rate was 500 Hz, maximum frequency was taken to be 250 Hz. Therefore 6-level decomposition was carried out using fourth-order Daubechies (db4) as the mother wavelet. It was selected as mother wavelet because it offers maximum correlation with the EEG signal. Thus the subbands were generated by 6-level decomposition using DWT (see Figure 3). Their relative energies (expressed as percentage of total energy) were calculated.

**Figure 5:**
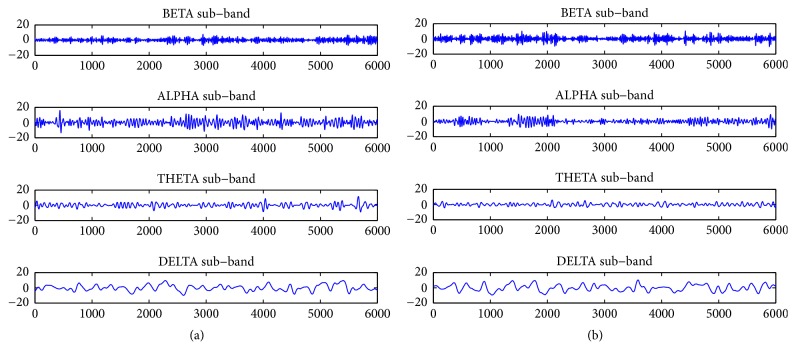
Various EEG subbands obtained using DWT: (a) normal EEG; (b) EEG in encephalopathy.

**Figure 6:**
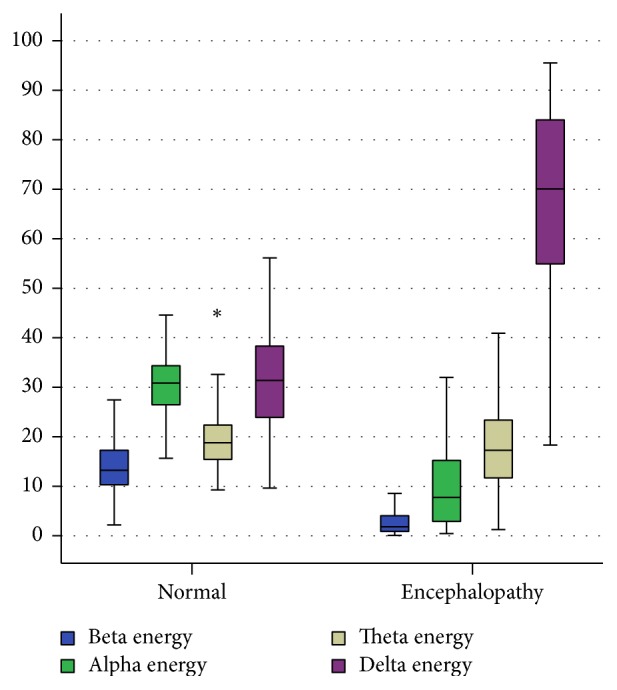
Distribution of EEG subband energies in encephalopathy and healthy groups.

This is similar to the result given by Kaplan [44]. The mean energy of the various subbands of EEG obtained from the encephalopathic patients and normal EEG after preprocessing and DWT are given in Figure 5. On analysis of the subband energies, it was found that the share of delta subband increased substantially with encephalopathy at the expense of decreased energy of predominant alpha waves; delta wave was seen to be around two times that of the normal EEG. See Table 1 and Figure 6 for details.

### 3.2. Statistical Analysis

Independent sample t-test was done to identify the subband energies which showed significant difference so that they can be potentially used for classifying between encephalopathy group and normal. Delta subband energy (*p* value <0.01;* t*= -25.06), alpha energy (*p* value <0.01;* t*= 32.31), and beta energy (*p* value <0.01;* t*=24.27) showed significant difference. Theta subband energy (*p* value >0.05;* t*=0.58) did not show significant difference between the two groups [*t *is the test statistic of independent sample t-test and p value shows its significance;* p *value less than 0.01 shows very high significance].

### 3.3. Classification Using Support Vector Machine (SVM)

We have implemented an SVM classifier for the diagnosis of encephalopathy based on the energies of subbands of EEG signal. We used a subset of data to train and subsequently the rest of the data were tested (see Table 2).

The features used for classification were

- energies of all subbands, i.e., delta, theta, alpha, and beta,
- energies of delta, alpha, and beta individually (as these were found to be significantly different in the statistical tests).

**Figure 7:**
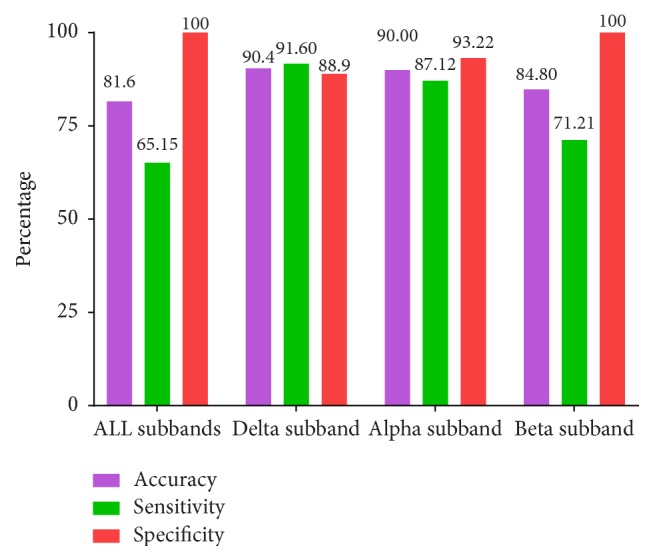
Performance parameters of SVM classifier based on different subband energies of EEG. Accuracy, sensitivity, and specificity are colour coded in the bar diagram.

Accuracy, sensitivity, and specificity are mainly used as performance parameters for the classifier. Sensitivity is the ability of the test to find out the diseased cases correctly (TP/TP+FN). Specificity is the ability of the classifier to find out the normal cases rightly (TN/TN+FP). Accuracy may be described as the ability of the classifier to distinguish diseased and normal cases correctly (TP+TN/TP+TN+FP+FN). Test statistics of the classifier are given in Table 3. The test showed highest performance when delta alone was used as the parameter with a sensitivity of 91.67% and specificity of 88.98% giving an accuracy of 90.4%. Performance parameters of the classifier for various feature sets are demonstrated in Figure 7. When alpha alone was used as the parameter the sensitivity was 87.12% and specificity was 93.22% giving an accuracy of 90%. This can be related to the phenomenon of loss of alpha activity and prominence of delta waves found in encephalopathy. The accuracy dropped when other combinations of subband energies were used as parameters.

Normal EEG background activity in an person who is awake is in the alpha range in the posterior head region. The initial EEG changes in encephalopathy are mild slowing of background which is reactive to external stimuli, followed by intermittent polymorphic delta activity. With worsening encephalopathy, there is continuous polymorphic delta activity persisting >80% of the record which is unreactive to external stimuli, with absent posterior dominant background [6]. Severe encephalopathy may progress to burst-suppression pattern (bursts of slow and sharp waves lasting 1-3 seconds, followed by suppression lasting 5-10 seconds), background suppression (<10 *μ*V), and electrocerebral silence (<2*μ*V). In summary, the EEG in encephalopathy is characterized by the loss of normal posterior dominant alpha background activity in the awake state with the appearance of delta waves, initially intermittent followed by continuous delta activity [19, 45]. Hence our finding of higher sensitivity and specificity of delta band and alpha band in encephalopathy is expected.

## 4. Conclusion

The “splitting” of an EEG waveform into its various frequency subbands maybe best performed using discrete wavelet transform compared with the customarily used frequency domain approach like fast Fourier transform. Our study concludes the relevance of wavelet decomposition in EEG analysis where time localisation of frequency components of the signal is possible. After applying LPF-TVD filtering, the EEG subbands were extracted using DWT and their energies were calculated. Statistical tests conducted revealed significant difference in delta, alpha, and beta between encephalopathy and normal EEG. Implementation of SVM classifier gave higher performance parameters for classifying the two groups when delta alone or alpha alone were taken as the features. The results correlate with the explanation of loss of normal alpha rhythm and prominence of delta rhythm during encephalopathy. This work can be extended for identifying various stages and severity of encephalopathy. This study provides a complete framework for the automated diagnosis of encephalopathy based on subband energies of EEG.

## Figures and Tables

**Table 1:** Values of mean, standard deviation, and standard error of subbands energies of normal and encephalopathic EEG.

	**Normal/ Encephalopathy**	**N**	**Mean**	**Std. Deviation**
**Beta energy (**%** of total energy)**	Normal	218	14.96	6.85
	Encephalopathy	232	2.89	2.71

**Alpha energy (**%** of total energy)**	Normal	218	30.60	6.03
	Encephalopathy	232	9.64	7.68

**Theta energy (**%** of total energy)**	Normal	218	19.71	6.47
	Encephalopathy	232	19.20	11.78

**Delta energy (**%** of total energy)**	Normal	218	31.39	10.34
	Encephalopathy	232	67.73	19.34

**Table 2:** Data set for training and testing.

	**Encephalopathy**	**Normal**	**Total**
**Training**	100	100	200

**Testing**	132	118	250

**Table 3:** Test statistics of SVM classifier based on different feature sets based on subband energies of EEG.

	**All sub-bands**	**Delta only**	**Alpha only**	**Beta only**
**True Positive (TP)**	86	121	115	94

**True Negative (TN)**	118	105	110	118

**False Positive (FP)**	0	13	8	0

**False Negative (FN)**	46	11	17	38

## Data Availability

As per the ethical committee guidelines of the institution, where the study was conducted, it is not permitted to share the EEG data and patients' confidential information in a public repository.
